# Retraction: An Overview of the Pathology and Emerging Treatment Approaches for Interstitial Cystitis/Bladder Pain Syndrome

**DOI:** 10.7759/cureus.r14

**Published:** 2019-04-16

**Authors:** Asad Ali, Nouman Safdar Ali, Muhammad Bilal Malik, Zohaib Sayyed, Malik Qistas Ahmad

**Affiliations:** 1 Internal Medicine, CMH Lahore Medical College and Institute of Dentistry, Lahore, PAK; 2 Internal Medicine, Allama Iqbal Medical College, Lahore, PAK; 3 Internal Medicine, Shifa College of Medicine, Lahore, PAK; 4 Pediatrics, Shaikh Khalifa Bin Zayed Al-Nahyan Medical and Dental College, Lahore, PAK; 5 Hematology/Oncology, University of Arizona Cancer Center, Tucson, USA

This article has been retracted at the request of the Editor-in-Chief as it contains significant plagiarism from Douglas-Moore JL, Goddard J. Current best practice in the management of cystitis and pelvic pain. *Therapeutic Advances in Urology*. 2018;10(1):17-22. 10.1177/1756287217734167

A key condition of article submission is that authors must explicitly declare that their work is original and has not appeared in a publication elsewhere. Re-use of any data must be appropriately cited. This duplication was not detected by Cureus plagiarism check software as much of the material had been rewritten just enough to avoid detection. While the authors have maintained that this was the result of working with a contract editor, they are ultimately responsible for the draft submitted.

As such this article represents a severe abuse of the scientific publishing system. The Cureus Journal of Medical Science takes a very strong view on this matter and we apologize that this was not detected during the submission process. The relevant author institutions and departments have been notified.

